# Methylsulfonylmethane Suppresses Breast Cancer Growth by Down-Regulating STAT3 and STAT5b Pathways

**DOI:** 10.1371/journal.pone.0033361

**Published:** 2012-04-02

**Authors:** Eun Joung Lim, Dae Young Hong, Jin Hee Park, Youn Hee Joung, Pramod Darvin, Sang Yoon Kim, Yoon Mi Na, Tae Sook Hwang, Sang-Kyu Ye, Eon-Soo Moon, Byung Wook Cho, Kyung Do Park, Hak Kyo Lee, Taekyu Park, Young Mok Yang

**Affiliations:** 1 Department of Pathology, School of Medicine, and Institute of Biomedical Science and Technology, Konkuk University Glocal Campus, Seoul, South Korea; 2 Department of Emergency Medicine, Konkuk University Hospital, Seoul, South Korea; 3 Department of Pharmacology, College of Medicine, Seoul National University, Seoul, South Korea; 4 Department of Internal Medicine, School of Medicine, Konkuk University Glocal Campus, Chung-Ju, South Korea; 5 Department of Animal Science, College of Life Sciences, Pusan National University, Busan, South Korea; 6 Genomic Informatics Center, Hankyong National University, Anseong, South Korea; 7 Department of Biotechnology, College of Biomedical and Health Science, Konkuk University Glocal Campus, Chung-Ju, South Korea; Sun Yat-sen University Medical School, China

## Abstract

Breast cancer is the most aggressive form of all cancers, with high incidence and mortality rates. The purpose of the present study was to investigate the molecular mechanism by which methylsulfonylmethane (MSM) inhibits breast cancer growth in mice xenografts. MSM is an organic sulfur-containing natural compound without any toxicity. In this study, we demonstrated that MSM substantially decreased the viability of human breast cancer cells in a dose-dependent manner. MSM also suppressed the phosphorylation of STAT3, STAT5b, expression of IGF-1R, HIF-1α, VEGF, BrK, and p-IGF-1R and inhibited triple-negative receptor expression in receptor-positive cell lines. Moreover, MSM decreased the DNA-binding activities of STAT5b and STAT3, to the target gene promoters in MDA-MB 231 or co-transfected COS-7 cells. We confirmed that MSM significantly decreased the relative luciferase activities indicating crosstalk between STAT5b/IGF-1R, STAT5b/HSP90α, and STAT3/VEGF. To confirm these findings *in vivo*, xenografts were established in Balb/c athymic nude mice with MDA-MB 231 cells and MSM was administered for 30 days. Concurring to our *in vitro* analysis, these xenografts showed decreased expression of STAT3, STAT5b, IGF-1R and VEGF. Through *in vitro* and *in vivo* analysis, we confirmed that MSM can effectively regulate multiple targets including STAT3/VEGF and STAT5b/IGF-1R. These are the major molecules involved in tumor development, progression, and metastasis. Thus, we strongly recommend the use of MSM as a trial drug for treating all types of breast cancers including triple-negative cancers.

## Introduction

Breast cancer (BC) is the major cancer affecting females in the United States. Additionally, more than 1 million women worldwide are diagnosed with this disease per year. BC is the second most common cause of cancer-related deaths with ∼400,000 patients dying due to this disease every year [1], [2]. This disease is the major cause of death in women between the ages 45 and 55 y [3]. Approximately, 15% of BCs are triple-negative breast cancer, a type that is more prevalent among young African, African-American, and Latino women [4]. This type of aggressive breast cancer has unique molecular profiles. This subtype is clinically negative about the expression of estrogen receptor (ER) and progesterone receptor (PR), and does not over-express human epidermal growth factor receptor-2 (Her-2) protein. No targeted therapies exist for treating TNBC, and this disease frequently displays distinct patterns of metastasis [3].

Human BC frequently expresses the epidermal growth factor (EGF) receptor. Human epidermal growth factor-2 (Her-2), -3, and -4, orphan receptors of the EGF receptor family, that are co-expressed with other EGF receptors. The proto-oncogene Her-2 is located on chromosome 17. In case of 25 – 30% breast cancers, Her-2 is over-expressed. Apart from this, over-expression of Her-2 has been reported in many other aggressive breast cancers [5]. Ligand binding activates these receptors so that they form homo/heterodimers and stimulate downstream signalling pathways. The Ras/Raf/MAPK and PI3-K/Akt pathways involved in cell proliferation, and survival are major targets of activated EGF receptors [6]. Her-2 over-expression has been shown to result in increased transformation, tumorigenicity, proliferation, and invasiveness [7].

Approximately one-half of primary breast tumors are ER^+^/PR^+^, whereas less than 5% are ER^−^/PR^+^ [8]. PR is a specific receptor that belongs to the superfamily of ligand-activated nuclear receptors [9]. PR exists in two isoforms, PR-A and PR-B; both are expressed in humans [10]. Both receptors bind progestins and promote epithelial cell proliferation as well as lobulo-alveolar development [11]. The binding of progesterone to PRs induces the formation of receptor homo- or heterodimers. This conformational change leads to increased receptor phosphorylation, and interaction with target gene promoters, specific co-activators, and general transcription factors [12]. PRs have some prognostic and predictive implications [13], [14]. Together with ERs, PRs make cells sensitive or resistance to different therapies [15]. Based on the expression pattern, PR breast cancer may be ER^+^/PR^+^ or ER^+^/PR^−^, and PR^+^ breast cancers have been found to be more differentiated than PR^-^ breast cancers [8].

High levels of estrogen receptor-α (ER-α) promote hormone-dependent tumor growth by converting the receptor as a ligand-dependent transcription factor. ER-α-dependent processes require different concentrations of receptors and is not always the factor limiting hormone responsiveness. In breast tumors, increased proliferation rates have been observed with high ER-α expression [16] and thymidine kinase activity [17]. The ER-α receptor and steroid hormones regulate vascular endothelial growth factor (VEGF) in breast cancer *in vivo* [8].

Vascular endothelial growth factor-A (VEGF-A) is considered to be the most important and potent pro-angiogenic factor involved in tumor growth [19]. The binding of VEGF to VEGFR induces conformational changes in the receptor followed by auto-phosphorylation of the receptor [20]. VEGF expression is regulated by hypoxia, steroid hormones, nitric oxide, and cytokines [21], [22].

Signal transducer and activator of transcription 3 (STAT3) is important for breast involution after weaning [23] and a prognostic factor for breast cancer [24]. We have reported that STAT3 modulates VEGF through HIF-1α [25]. Tumor angiogenesis is enhanced as VEGF expression is up-regulated by increased STAT3 activity. In addition, decreased Src-induced VEGF expression is observed when Stat3 signaling is blocked [26]. This indicates that Stat3 represents a common anti-angiogenesis target for blocking multiple signaling pathways in human cancers. Another important member of the STAT family, STAT5b, regulates growth, differentiation, and survival of mammary and solid tumors. Recently, we reported that STAT5b regulates the transactivation of cyclin D1 and IGF-1 upon hypoxia stimulation in breast cancer cells [27], [28], [29].

Methylsulfonylmethane (MSM) is a very simple organic sulfur-containing compound with a molar mass of 94.13 g/mol. MSM contains only eleven atoms and is found in foods, including fruits, vegetables, grains, and beverages [30]. It is a symmetric molecule with no isomeric forms. Dimethyl sulfone has also been detected in the human brain [31], blood plasma, and cerebrospinal fluid [32] by proton magnetic resonance spectroscopy. MSM is volatile, easily lost during cooking, and is believed to be non-toxic [33], [34]. MSM decreases arthritis pain and improves physical function of osteoarthritis human knees without major adverse events [35]. This compound has also been found to be effective for treating allergies [36], osteoarthritis pain [35], inflammation [37], repetitive stress injuries [38], and bladder disorders like intestinal cystitis [39]. MSM can induce wound healing, contact inhibition, and can block the ability of cells to migrate through the extra-cellular matrix. Furthermore, it can restore anchorage-dependent growth and irreversible senescence followed by arborization with melanosomes in arbors seen in murine melanoma cell lines [40].

In this study, we proposed that MSM suppresses tumor growth via inhibition of the STAT3 and STAT5b pathways. To test this hypothesis, we investigated the effects of MSM on human breast cancer cells and in the experimental animal model. The effects of MSM on the expression of STAT3, STAT5b, and their downstream targets were analysed. From the results obtained, we found that MSM down-regulates triple-negative hormone receptor expression in hormone-responsive cell lines and suppresses the growth of breast cancer xenografts through its multi-targeted action.

## Materials and Methods

### Ethics Statement

All procedures for animal experiment were approved by the Committee on the Use and Care on Animals (Certificate No: KUB00313, Institutional Animal Care and Use Committee, Seoul, Korea) and performed in accordance with the institution guidelines.

### Materials

Methylsulfonemethane (MSM) was purchased from Fluka/Sigma Co. (St. Louis, MI).

### Antibodies and Reagents

Dulbecco’s modified eagle’s medium (DMEM), DMEM/F-12, RPMI 1640, 10% fetal bovine serum (FBS) and trypsin-EDTA were purchased from Gibco-BRL (GrandIsland, NY). L-15 medium, anti-actin antibody, insulin and EGF were obtained from Sigma Chemical (St. Louis, MO). Anti- STAT5b antibodies, secondary antibodies (goat anti-mouse IgG-horseradish peroxidase) were obtained from Santa Cruz Biotechnology (Santa Cruz, CA). Anti-phospho-STAT3(Tyr705) and Anti-phospho-IGF-1R(Tyr1131) antibodies were obtained from cell signaling (Beverly, MA). Anti-phospho-STAT5b(Tyr699) antibodies was obtained from upstate (Lake Placid, NY). The secondary antibody (horseradish peroxidase-conjugated donkey anti-rabbit IgG), the enhanced chemiluminescence (ECL plus) detection kit was purchased from Amersham Pharmacia Biotech. (Piscataway, NJ). Restore^TM^ Western Blot Stripping Buffer and NE-PER kit were purchased from Pierce (Rockford, IL). The luciferase assay substrates, reporter lysis buffer, and electrophoretic mobility shift assay (EMSA) kit were purchased from Promega Corp. (Madison, WI). FuGene 6 transfection reagent was from Roche (Basel, Switzerland), RNeasy mini kit and Qiaprep spin miniprep kits were purchased from Qiagen (Germany).

### Cell Culture

MCF-10A (kind gift from Dr. Ssang-Goo Cho, Konkuk University, Korea), immortalized normal human breast epithelial cells were grown to confluency in phenol red free DMEM/F2 medium supplemented with cholera toxin (20 µg/mL), insulin (10 mg/mL), EGF (250 µg/mL), 1% penicillin/streptomycin, and horse serum (5%). MCF-7 (No: 30022, KCLB, Korea), T-47D (No: 30133, KCLB, Korea) and SK-BR3 (No: 30030, KCLB, Korea), human breast cancer cells, were grown to confluency in RPMI 1640 medium containing 10% FBS, insulin (5 • g/ml), and EGF (10 ng/ml). COS-7 (No: 21651, KCLB, Korea), monkey kidney cells, and MDA-MB 231 (No: 30026, KCLB, Korea), human breast cancer cells, were cultured in DMEM containing 10% FBS, 2 mM glutamine, and 100 U/ml penicillin and streptomycin at 37^°^C in 5% CO_2_. At the start of each experiment, the cells were resuspended in the medium at a density of 2.5×10^5^ cells/ml.

### MTT assay

Cell viability was assayed by measuring blue formazan that was metabolized from 3-(4,5-dimethylthiazol-2-yl)-2,5-diphenyl tetrazolium bromide (MTT) by mitochondrial dehydrogenase, which is active only in live cells. One day before drug application, cells were seeded in 96-well flat-bottomed microtiter plates (3,000–5,000 cells/well). Cells were incubated for 24 h with various concentrations of MSM. MTT (5 mg/ml) was added to each well and incubated for 4 h at 37°C. The formazan product was dissolved by adding 200 μl dimethylsulfoxide (DMSO) to each well, and the plates were read at 550 nm. All measurements were performed in triplicate, and each experiment was repeated at least three times.

### Apoptosis Analysis

Fluorescein-conjugated Annexin V (Annexin V-FITC) was used to quantitatively determine the percentage of cells undergoing apoptosis. Treated cells were washed twice with cold PBS and then resuspended in binding buffer at a concentration of 1x10^6^ cells/ml. Five microliters of Annexin V-FITC and 10 µl of propidium iodide were added to suspended cells. After incubation for 15 min at room temperature in the dark, the percentage of apoptotic cells was analyzed by flow cytometry (Becton-Dickinson FACScan, San Jose, CA). For positive controls 10 µM camptothecin and 23 µM actinomycin D were used.

### Total Cell Lysis

Breast cancer cells were treated with MSM for determined times. Cells were lysed on ice for 10 min in radioimmunoprecipitation assay (RIPA) lysis buffer containing protease and phosphatase inhibitors. Cells were disrupted by aspiration through a 23-gauge needle, and centrifuged at 15,000 rpm for 10 min at 4°C to remove cellular debris. Protein concentations were measured using the Bradford method.

### Western Blot

Whole cell extracts (WCE) from breast cancer cells were prepared by described previously and quantified using Bradford’s method. Equal amounts of protein obtained by total lysis were subjected to 10% SDS-PAGE and electrophoretically transferred onto a nitrocellulose membrane. The blots were blocked with 5% skim milk or BSA in TBS-T buffer. It was then incubated overnight with primary antibody followed by washing with TBS-T and incubation with secondary antibody (anti-mouse or anti rabbit IgG HRP conjugate, 1∶1,000 dilutions with skim milk or BSA). Detection was done by using enhanced chemiluminescence (ECL plus) detection kit.

### Reverse Transcription Polymerase Chain Reaction (RT-PCR)

Total RNA was isolated from the cells by using Tri reagent (Sigma Chemical Co., St. Louis, MO) and quantitated spectrophotometrically at 260 nm. RT-PCR analysis for VEGF, IGF-1R and 18s RNA was performed (Table S1). Briefly, 1 µg of RNA was reverse transcribed, and nested PCR was carried out by using 2 µl of cDNA. The PCR condition consisted of denaturation for 1 min at 94°C, annealing for 1 min at 58°C, and extension for 1 min at 72°C. RT-PCR products were analyzed on 1% agarose gel stained with ethidium bromide.

### Electrophoretic Mobility Shift Assay (EMSA)

STAT5 and STAT3 DNA binding activity was detected using an electrophoretic mobility shift assay (EMSA), in which a labeled double-stranded DNA sequence was used as a DNA probe to bind active STAT5b and STAT3 protein in nuclear extracts. Nuclear protein extracts were prepared with the Nuclear Extract Kit (Panomics, AY2002). EMSA experiment is performed by incubating a biotin-labeled transcription factor (TF-STAT5 and STAT3) probe with treated and untreated nuclear extracts.

### Co-transfection and Luciferase Assay

The expression vectors for mouse STAT5b (pMX/STAT5b; kindly provided by Dr. Koichi Ikuta, Kyoto University, Japan) were constructed as previously described. cDNA for STAT5b was inserted into the *EcoR*?and *Sal*?sites of the pMX vector. IGF-1R (kindly provided by Dr. Haim Werner, Tel Aviv University, Israel) genomic DNA fragments, including nucleotides -2350 to +640 (nucleotide 1 corresponds to the transcription start site of the rat IGF-1R gene), was sub-cloned upstream of a promoterless firefly luciferase reporter in the pGL2P vector (Promega, Madison, WI). For reporter gene assays, COS-7 cells were transiently co-transfected with the plasmid pGL2P, IGF-1R or HSP90α (kindly provided by Dr. Carrie Shemanko, Calgary University, Canada) construct and the STAT5b expression vector.

Cells were co-transfected with various combinations the following constructs; wild-STAT3 (gifts from Dr. Shong, Chungnam National University, Korea); the VEGF reporter construct containing 2.7 kb of the VEGF promoter region. Transfected cells were washed with ice-cold PBS, lysed, and lysates were used directly to measure luciferase activity. The luciferase activity of each sample was determined by measuring luminescence for 10 s on a Lumat LB 9507 luminometer (EG&G Berthold, Oak Ridge, TN). The experiments were performed in triplicate, and similar results were obtained from at least three independent experiments.

### Live Cell Microscopy

Cells were plate on 6 well culture dishes and incubated at 37°C, 5% CO_2._ Time series (10 min) of phase contrast images were acquired at a video rate of 1 frame/5s with a Real-time cell observer (Carl Zeiss). Time series of cells with and without methylsulfonylmethane were obtained at 0-120 min after adding the compound and every 24 h for up to four days.

### Tumorigenicity

All procedures for animal experiment were approved by the Committee on the Use and Care on Animals (Institutional Animal Care and Use Committee, Seoul, Korea) and performed in accordance with the institution guidelines. MDA-MB 231 tumor xenograft were established by subcutaneously inoculating 1x10*^7^* cells into the right flanks of 5-week-old Balb/c nude mice (Orient Bio, Seongnam-Si, Korea). When tumors reached between 6 to 8 mm in diameter, mice were randomly assigned to control group, MSM 3%-treated group and MSM 5%- treated group respectively with 6 mice in each group. The drug was administered as intragastric injections of 100 μl, containing 3% MSM or 5% MSM in triple distilled water. The injections were repeated one time every other day. Tumor growth was monitored by periodic measurements with calipers. Tumor volume was calculated using the formula: tumor volume (mm^3^) = maximal length (mm) × (perpendicular width) (mm^2^)/2. Animals were sacrificed when the diameter of tumors reached 2 cm or after 30 days of treatment. In our experiments, no mice were observed to be died of tumor loading. All available human breast cancer xenograft collected from mice were reviewed and included in the study.

### Real-time Polymerase Chain Reaction

Total RNA was isolated from tumor xenograft and quantified by a spectrophotometric analysis at 260 nm. The cDNA synthesis and the probe used for the detection of IGF-1 and β-actin from a TaqMan gene expression assay kit (Applied Biosystems Inc.). PCR was monitored in real time using the ABI Prism 7900 HT Real time PCR System (Applied Biosystems Inc., CA).

### Immunohistochemistry

Formalin-fixed paraffin-embedded breast tumor xenografts were sliced into 5 μm thick section. These sections were deparaffinized with 100% xylene, rehydrated with decreasing concentration of ethyl alcohol, permeabilised with 0.1% triton X-100 and blocked with 10% NGS (Nomal Goat Serum in PBS). These were then incubated with the STAT5b, IGF-1R, STAT3 and VEGF antibody followed by incubation with the secondary antibody, Alexa Fluor 488 (rabbit) and Alexa Fluor 594 (mouse) (Invitrogen). For the detection of nuclear level, tissue sections were incubated on DAPI for one minute and rinsed with PBS. Samples were observed and photographed under the fluorescent microscope.

### Data analysis and Statistics

The results of the experiments are expressed as mean ± SEM. Statistical analysis was done by t-tests or ANOVA-tests using the SAS program.

## Results

### Cytotoxicity of MSM in Human Breast Cancer Cell Line

**Figure 1:**
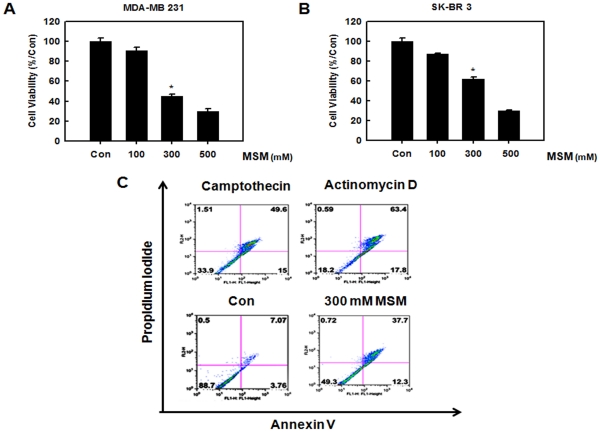
MSM induced cytotoxicity in human breast cancer cell lines in a dose-dependent manner. The cytotoxicity is confirmed as apoptosis through flow cytometry. A, effect of MSM on triple-negative MDA-MB 231 cells. B, effect of MSM on SK-BR3 cells. C, flow cytometry of MDA-MB 231 cells using Annexin V-FITC, propidium iodide flow cytometry.

To determine the effect of MSM on cell survival, human breast cancer cell lines MDA-MB 231 and SK-BR3 were exposed to different concentrations of MSM (100, 300, and 500 mM) for 24 h. The number of MSM treated cells during the logarithmic phase of growth was compared with that of the control cells. MDA-MB 231 cell growth was inhibited by ∼55% with 300 mM MSM and ∼70% with 500 mM MSM (Fig. 1A). SK-BR3 cell growth was inhibited by ∼38% with 300 mM MSM and ∼70% with 500 mM MSM (Fig. 1B). Thus, treatment with MSM substantially decreased the viability of MDA–MB 231 and SK-BR3 cells in a dose-dependent manner.

### MSM-induced Apoptosis in MDA-MB 231 Cells

MTT assay on MDA-MB 231 and SK-BR3 showed that MSM had high levels of cytotoxic activity. To differentiate this from necrosis and to confirm it as apoptosis, we performed fluorescein-conjugated annexin V (annexin V-FITC) flow cytometry. We quantitated the number of cells undergoing apoptosis. Our results showed that 300 mM of MSM induced apoptosis in 50% of the MDA-MB 231 cells (Fig. 1C). The positive control camptothecin (10 µM) and actinomycin D (23 µM) induced apoptosis approximately 65% and 81% respectively.

### Expression of Stats and Triple-negative Receptors are down-regulated by MSM

**Figure 2:**
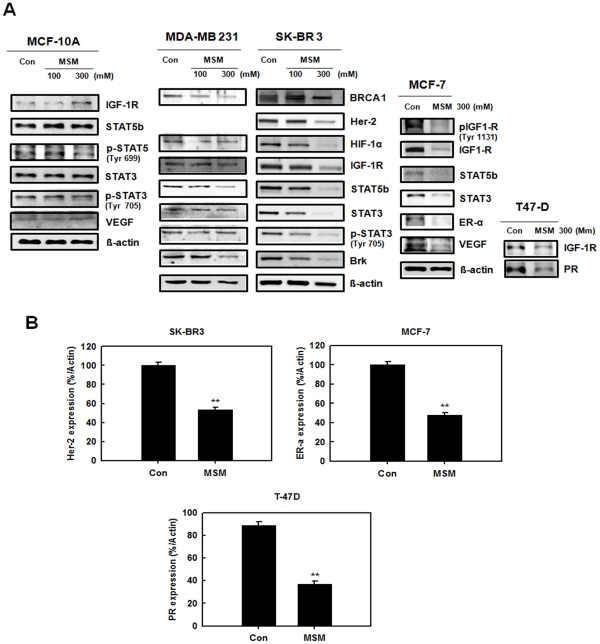
A, change in expression of STATs, IGF-1R, VEGF, BRCA, Brk, and hormone receptors in human breast cancer cells by MSM. MSM substantially decreased the protein expression of human breast cancer cells in a dose-dependent manner. Whereas in normal cell line MCF-10A (Panel 1), MSM did not alter the protein expression levels. B, expression of Her-2, ER-α and PR by MSM in human breast cancer cells SKBR-3, MCF-7, and T-47D. The values are means ± S.E (n = 3) after normalization to β-actin levels (internal control). Asterisks indicate a statistically significant decrease by t-test (**p<0.01). Cell lysates were separated by 10% SDS–PAGE and transferred to a nitrocellulose membrane. The membrane was blotted with the primary antibody, then stripped and reprobed with the next antibody and so on. Data are one representative of three independent experiments.

The expression of STAT proteins and triple-negative hormone receptors was down-regulated in response to MSM in a dose-dependent manner in non-aggressive tumor cells like SK-BR3, MCF-7, and T-47D (Fig. 2A). In MDA-MB 231 cells expression of STAT proteins decreased. The expression of IGF-1R decreased in the entire breast cancer cell lines treated with 300 mM of MSM whereas it found unaltered in normal cell line MCF-10A. This was also observed for STAT5b and STAT3. The STAT5 phosphorylation level also found unaltered by MSM in MCF-10A cells. The expression of triple-negative hormone receptors, Her-2, ER-α, and PR as well found to be suppressed by 300 mM of MSM (Fig. 2B).

### Igf-1R and Vegf mrna Expression are Down-regulated by MSM

**Figure 3:**
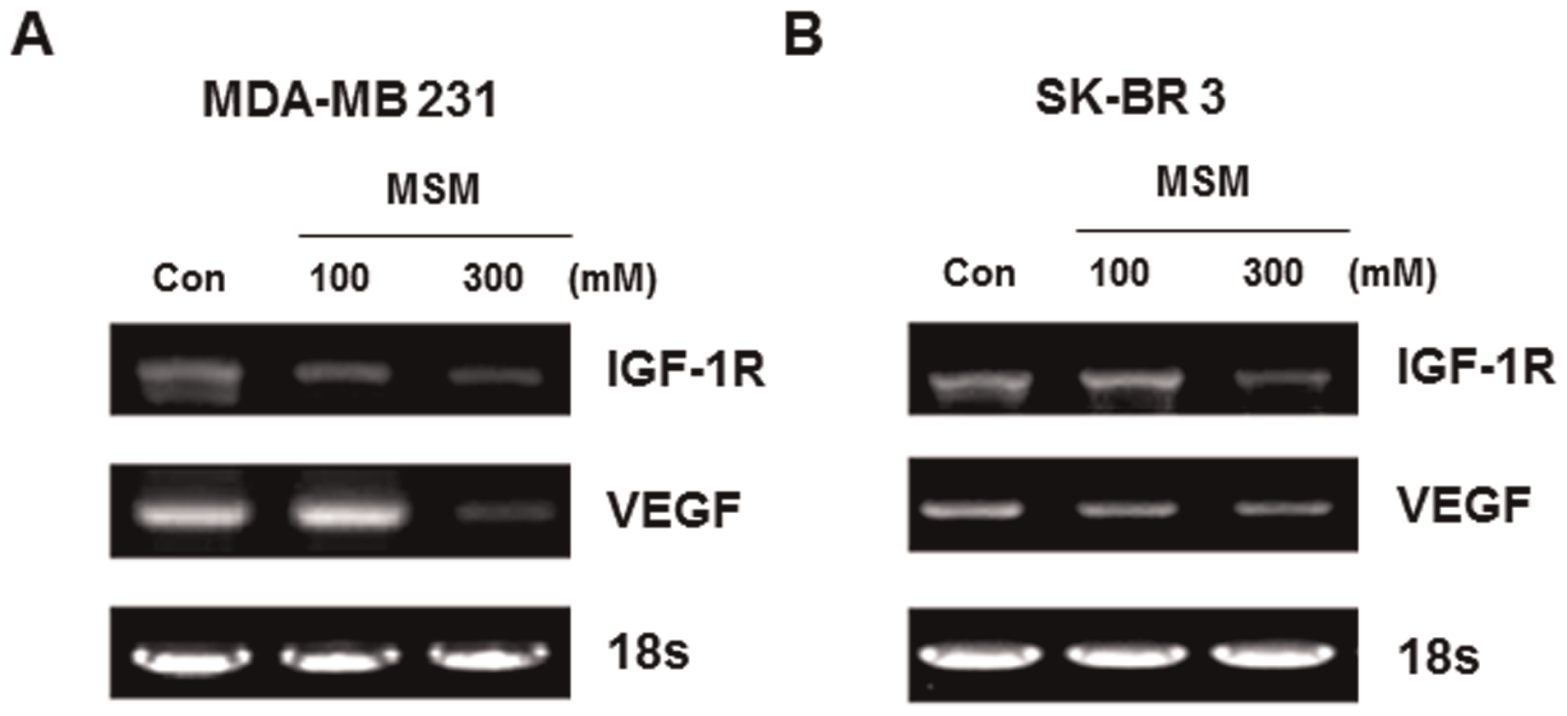
RT-PCR analysis of breast cancer cell lines revealed MSM can effectively down regulate the expression of IGF-1R and VEGF in dose dependent manner. A, IGF-1R and VEGF level in MDA-MB 231 cells. B, IGF-1R and VEGF level in SK-BR3 cells (table 1).

Rt-pcr for both MSM-treated and untreated cells yielded amplified products of 312 and 522 bp, which corresponds to VEGF and IGF-1R mRNA, respectively. igf-1r and vegf were amplified using gene-specific primers. The expression of both igf-1r and vegf was down-regulated in a dose-dependent manner by MSM (Fig. 3). 18S expression (control) was unaffected by MSM regardless of the concentration.

### MSM Inhibited the Binding of STAT5 to the IGF-1R and STAT3 to the VEGF Promoter sites

**Figure 4:**
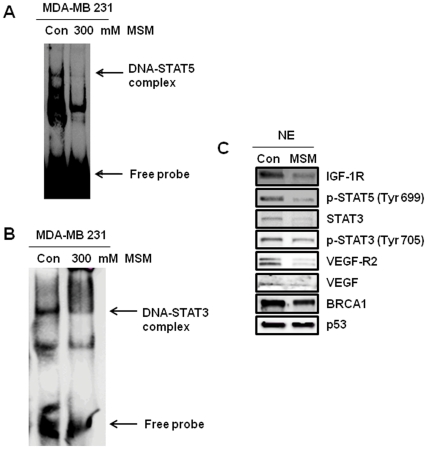
DNA binding activities of STAT5 to IGF-1R and STAT3 to VEGF sites in transfected MDA-MB 231 cells were detected by EMSA. A, binding activity of STAT5b to the STAT5 binding site (GAS-1) of IGF-1 promoter. B, binding activity of STAT3 on the VEGF promoter site. C, nuclear protein extracts separated and blotted onto nitrocellulose membrane showing decrease in the level of p-STAT3 and p-STAT5.

MSM inhibited the binding of STAT5 to the IGF-1R site and suppressed STAT3 binding to the VEGF promoter sites. MDA-MB 231 cells were treated with 300 mM MSM. As shown in Fig. 4A, no DNA binding activity was found in the presence of 300 mM MSM. The nuclear extract showed decreased level of p-STAT5 (Fig. 4C). Hence, MSM inhibited the phosphorylation of STAT5b to p-STAT5, and binding to the promoter sites of IGF-1R. Moreover, decreased binding of STAT3 to VEGF promoter sites was detected (Fig. 4B), with very low expression levels of STAT3 (Fig. 4C). Figure 4C apparently shows the expression of prominent metastatic receptor VEGF-R2 blocked near completely, whereas the expression of tumor suppressor protein p53, and BRCA-1were maintained.

### MSM Inhibited STAT5b/IGF-1R, STAT5b/HSP90α, and STAT3/VEGF Promoter Activities

**Figure 5:**
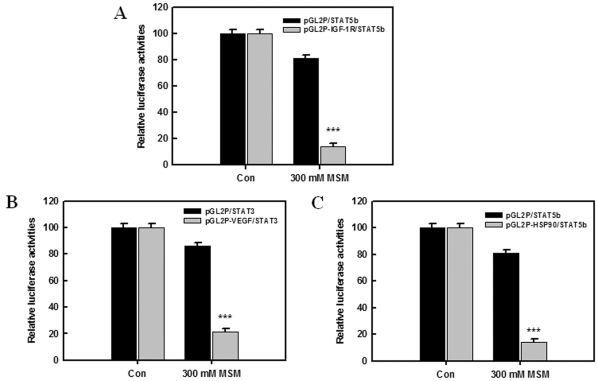
Activation of the STAT3/VEGF promoter related pathway in COS-7 cells by MSM. A, change of STAT5b/IGF-1R promoter. B, change of STAT5b/HSP90α promoter and C, change of STAT3/VEGF promoter. Data represents means of at least three separate experiments. Asterisks indicate a statistically significant decrease by ANOVA-test (***p<0.001).

The involvement of MSM in STAT3 mediated activation of the VEGF promoter was examined using the STAT3-deficient cell line, COS-7. The transcriptional effects of MSM on STAT5b/IGF-1R, STAT5b/HSP90α, and STAT3/VEGF were determined with a luciferase reporter assay. Fig. 5A, B and C show the relative luciferase activities of STAT5b/IGF-1R, STAT5b/HSP90α, and STAT3/VEGF, respectively. After 24 h of MSM (300 mM) treatment, relative luciferase activity was decreased and found to be statistically significant for STAT5b/IGF-1R, STAT5b/HSP90α and STAT3/VEGF (***P <0.001). These results suggest that STAT5b is a critical mediator of the IGF-1R, and STAT3 is of the VEGF pathway. It also confirms the critical role of MSM in inhibiting the promoter activities of STAT5b and STAT3, there by inhibiting the STAT5b and STAT3 signalling cascades.

### MSM Induced Migration Inhibition in Metastatic Human Breast Carcinoma Cells

**Figure 6:**
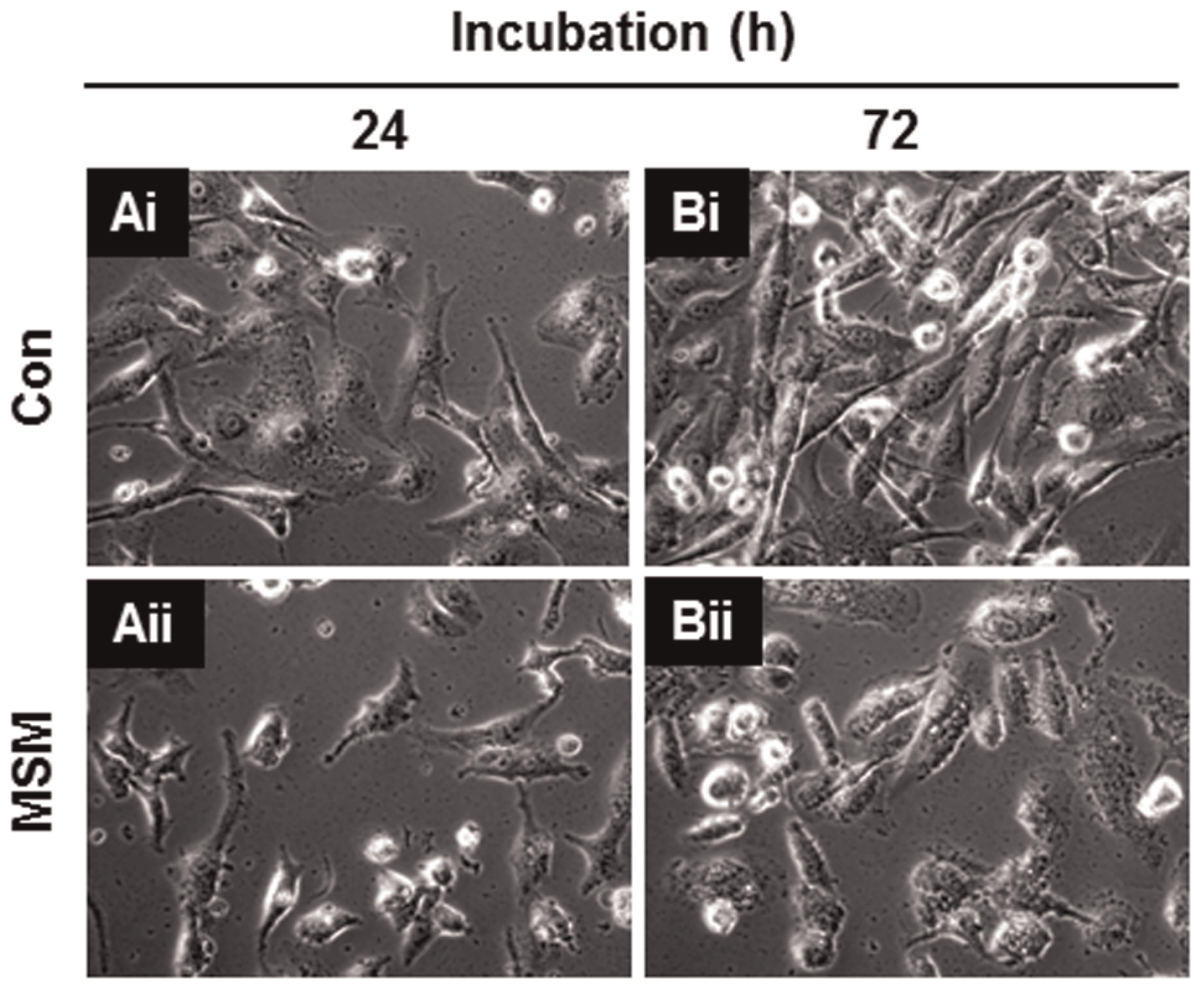
Methylsulfonylmethane induced migration inhibition in metastatic breast cancer cell line MDA-MB 231. **MDA-MB 231 cells (10^5^ cells/ml) were plated into DMEM medium.** After 24 h, medium was replaced with DMEM with and without MSM. Ai, MDA-MB 231 cells without MSM. Live cell image acquired after 24 h Aii, MDA-MB 231 cells in 200 mM MSM. Live cell image acquired after 24 h. Bi, MDA-MB 231 cells without MSM. Live cell image acquired after 72 h showing the morphological changes like arborization. Bii, MDA-MB 231 cells in 200 mM MSM. Live cell image acquired after 72 h showing no morphological alteration.

According to the cytotoxic studies, we confirmed the IC50 dosage of MSM as 300 mM (Fig. 1A). For getting the maximum number of viable cells, we reduced the concentration of MSM to 200 mM. In this concentration, MSM induced cytotoxicity up to 30% of MDA-MB 231 cells and 70% of MDA-MB 231 cells remained viable (Data not shown). This gave us an opportunity to decipher the actions of MSM. Live cell video microscopy demonstrated that metastatic breast cancer cells in 200 mM MSM stopped migrating through the adjacent layers of metastatic cells. The live cell microscopy also showed high morphological alterations in the control cells on continuous incubation for 72 h with a media change in every 24 h. After 72 h incubation, the morphology of control cells changed to sharp finger-like structures (Fig. 6Bi) resembling the actin filaments which are necessary for the migration. However, the 200 mM MSM treated cells remained without any morphological alterations (Fig. 6Bii). Moreover, the result clearly shows the cells grown under the absence of MSM (control) migrate under and over neighboring cells (Fig. 6Bi; Movie S1) whereas the MSM treated cells lost its ability for migration (Fig. 6Bii; Movie S2). Apart from this, the result gave an additional evidence of the ability of MSM to control the cell proliferation and apoptosis (Fig. 6).

### MSM Suppressed the Growth of Human Breast Cancer Xenografts

**Figure 7:**
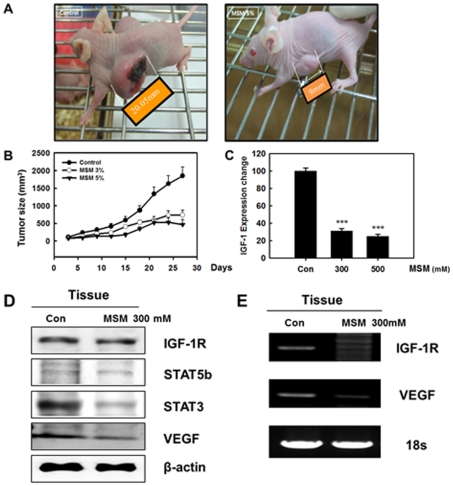
Breast cancer xenografts were established in mice by subcutaneous injection of MDA-MB 231 cells (1X10^7^ cells per mouse). A, image of tumor-xenografted nude mice model at the end of the treatment. B, tumor size growth curves during the treatment calculating the volume size of individual tumors. C, real time PCR results of IGF-1 mRNA from breast cancer xenografts. The real time PCR was performed using TaqMan Gene Expression assays for human IGF-1 and β-actin mRNAs from ABI on ABI 7900 HT Real-time PCR system. The values are means±S.E (n = 3) after normalization to β-actin mRNA levels (internal control). Asterisks indicate a statistically significant decrease by ANOVA-test (***p<0.001). D, regulation of protein expression in xenografts by MSM. E, RT-PCR analysis of IGF-1R and VEGF mRNA levels in xenografts showed the transcriptional regulation by MSM.

The *in vivo* tumor suppressive activity of MSM was evaluated in Balb/c mice bearing breast tumors induced by MDA-MB 231 cells (1x10^7^ cells/mL). Three separate groups of animals were studied, and MSM treatment started 2 weeks after the injection of human breast cancer cell line (MDA-MB 231). After 30 days of treatment, the diameter of tumor mass was measured. We observed a dose-dependent suppression of tumor growth (Fig. 7A). A graph was created showing the increase in tumor size with respect to the time of MSM treatment in days. The rate of tumor growth in the control group was significantly greater than that of the other two groups (Fig. 7B). The animals were sacrificed, and the xenografts were collected for further analysis.

### IGF-1 Expression Changes in Xenografts by MSM

**Figure 8:**
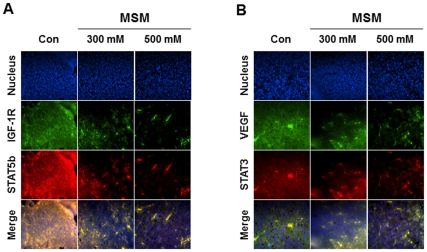
Immunohistochemistry studies confirmed MSM down regulates the expression of STAT5b, IGF-1R, STAT3, and VEGF. The xenografts were sliced to 5 µm thickness and treated with primary antibodies specific for STAT3, STAT5b, VEGF, IGF-1R, and detected using secondary antibody, Alexa Fluor 488 (rabbit) and Alexa Fluor 594 (mouse) using a magnification of 400X. A, IHC specific for STAT5b/IGF-1R. Results obtained clearly shows the decrease in STATb/IGF-1R level with no much alteration in the nucleus level. B, IHC specific for STAT3/VEGF. Results obtained clearly shows the decrease in STAT3/VEGF level with fewer alterations in the nucleus level.

The xenografts were subjected to Real Time-PCR analysis specific for IGF-1. The results showed that MSM inhibited the expression of IGF-1 in the *in vivo* system (Fig. 7C). A statistically significant reduction of IGF-1 expression was found in tissues from mice treated with 300 mM MSM (***P <0.001). Protein expression studies of the tissue produced results similar to the ones obtained from the *in vitro* analysis. The expression STAT5b, STAT3, VEGF, and IGF-1 decreased while IGF-1R levels were maintained (Fig. 7D). Down-regulation of VEGF expression by MSM was confirmed in the tumor xenografts by RT-PCR analysis (Fig. 7E). Immunohistochemistry specific for VEGF/STAT3 and IGF-1R/STAT5b confirmed the ability of MSM to down-regulate the expression of these factors (Fig. 8A and B). All of these results complimented the one we obtained from the *in vitro* analysis.

## Discussion

Cancer cells become resistant to different therapies over time; thus, it is necessary to target multiple signaling points for effective therapy. We have determined that MSM can inhibit STAT3/VEGF and STAT5b/IGF-1R pathways, thereby suppressing the growth of solid tumors. STAT3 is involved in tumor progression by inducing angiogenic factors such as VEGF [26]. VEGF, particularly VEGF-A, is considered to be the most important and potent pro-angiogenic factor involved in tumor growth [19]. Anti-angiogenic therapy appears to be a too narrow approach for treating patients with cancer. Cancer cells may find compensatory pathways for survival and metastasis. A combinatorial approach should produce better results under such conditions. MSM was found to have high levels of cytotoxic activity (Fig. 8) and anti-angiogenic activity by suppressing VEGF. This compound can halt tumor progression by blocking STAT5b and IGF-1R.

To evaluate the anti-cancer effects of MSM, we performed MTT assay using different concentrations of MSM. The results shown in Fig. 1A and 1B indicated that MSM exerted good cytotoxic effects on metastatic human breast cancer cells (MDA-MB 231) and comparatively less aggressive human breast cancer cells (SK-BR3). The IC_50_ was found to be 300 mM. MSM is an edible natural organic compound present in many food items and is not associated with any toxic effect even at higher concentration [33], [34]. Because of this, we used high concentration of the drug (300 mM) for further studies. Cytotoxicity resulting from MSM was confirmed as apoptosis by annexin V-FITC flow cytometry (Fig. 1C). These results were similar to the ones from a study by Caron *et al.* on a metastatic murine melanoma cell line. In their report, the authors state that 200 mM MSM exhibits anti-metastatic and anti-cancer activity. This finding was the first report on metastatic anti-cancer effect of MSM [40]. In addition to this, MSM may have dual function with anti-oncogenic effects and active-tumor suppressing effects in the nuclear level of cancer cells (Fig. 4C).

Anti-angiogenic studies of MSM in human breast cancer cells were mainly focused on VEGF and its regulator STAT3. Western blotting studies, DNA binding studies, luciferase assays, and transcriptional level regulation studies were performed to analyze the regulatory effects of MSM on STAT3 and VEGF. All results showed that MSM can effectively control the expression and activation of STAT3 and VEGF. The STAT nuclear translocation and DNA binding activity are generally not affected by STAT serine phosphorylation, but they are affected by STAT tyrosine phosphorylation [41]. Our study showed that MSM has the capacity to regulate the phosphorylation of STAT3 and STAT5b (Fig. 4C) and there by the remaining signaling cascades it mediates too.

STAT5 up-regulates genes encoding apoptosis inhibitors and cell cycle regulators, including Bcl-xL, cyclin D1, and p21 thus leading to oncogenesis [42], [43]. STAT5 has a major role in the development, prognosis, prediction, and progression of breast cancer [44]. In normal individuals, STAT5 is necessary for the terminal differentiation of mammary glands and lactogenesis [45]. Novel functions of STAT5b, a new target for breast tumor kinase/protein tyrosine kinase 6 (Brk/PTK6), have been reported [46] and the involvement of STAT5b in breast cancer cell migration has been demonstrated [47]. We previously reported that STAT5b may mediate the transcriptional activation of IGF-1 and cyclin D1 after hypoxic stimulation in human breast cancer cells [27], [29], [48]. Cyclin D1 has been shown to be regulated by STAT5b [49]. Cyclin D1 and IGF -1R are the key regulators of cell proliferation that are overexpressed in most breast cancers [50]. Furthermore, IGF-1 is a potent mitogen in cancer cells and plays an important role in tumorigenesis and tumor progression in a variety of cancers [51], [52].

The regulatory effects of MSM on tumorigenesis and tumor progression were studied by examining STAT5b/IGF-1R. Western blotting studies showed that MSM can effectively down-regulate the expression of STAT5b as well as IGF-1R in both aggressive and non-aggressive human breast cancer cell lines. IGF-1R blockage using different approaches leads to dramatic reductions in proliferation and other neoplasia parameters [53], [54]. Brk/PTK6 and HIF 1-α were also down-regulated by MSM (Fig. 2), showing the multiple target action of this compound for tumor suppression. RT-PCR and DNA binding studies showed that IGF-1R expression was down-regulated. MSM suppressed the activation of STAT5 and subsequent decreased DNA binding activity was observed in STAT5/IGF-1R (Fig. 4). Luciferase assay confirmed this finding by showing reduced activity of STAT5b/IGF-1R (Fig. 5).

The morphological alterations happening to MDA-MB 231 cells were observed in the presence and absence of MSM with the aid of live cell microscopy. According to the cytotoxic studies, we confirmed the IC_50_ dosage of MSM as 300 mM (Fig. 1A). To decipher further actions of MSM, we needed more live cells. So we reduced the concentration of MSM to 200 mM. In this concentration ∼70% of MDA-MB 231 cells remained viable, and this concentration used for live cell microscopic analysis. The analysis performed for 72 h with media change in every 24 h. The results obtained from live cell microscopy were surprising. MSM inhibited the migration of metastatic breast cancer cell lines (Movie S2). The cells which were not treated with MSM showed migration over and under the neighboring layers (Movie S1). Moreover, the control cells showed arborized morphology (Fig. 6Bi), whereas the 200 mM MSM treated cells were remained without any morphological alteration (Fig. 6Bii). These sharp protrusions were believed as actin filaments, which were needed for the migration of metastatic cells. The same result was observed in Caron’s paper on metastatic murine melanoma cell line. In their report, the authors state that 200 mM MSM induced contact inhibition and migration inhibition in metastatic melanoma cell line [40].

Triple-negative hormone receptors are very important in human breast cancer because they can make tumors sensitive or resistant to chemotherapy [15]. The mortality rate of patients with triple-negative breast cancer is very high [55]. These hormonal receptors are usually overexpressed in human breast cancers [56]. Therapy is usually designed based on the receptors responsible for the malignancy. The remarkable ability of MSM is that this compound can manage all types of malignancies associated with hormone receptors. Western blotting studies on hormone responsive breast cancer cells showed that MSM had the ability to down-regulate the expression of triple-negative hormone receptors (Fig. 2). In case of triple-negative cancer cells (MDA-MB 231), tumor progression is controlled by modulating IGF-1R. MSM can be used to treat herceptin-resistant breast cancers because the resistance is induced by IGF-1R [57] and MSM effectively controlled the expression of IGF-IR. So we hypothesized that MSM can also be used as a trial drug for tumors over expressing Her-2.

We performed *in vivo* studies to confirm the tumor suppressor activity of MSM and to propose its use as a trail drug for metastatic breast cancer therapy, especially for triple-negative breast cancer. MDA-MB 231 cells were injected subcutaneously into Balb/c nude mice for the induction of xenografts. After 30 days of treatment with MSM, these xenografts were removed and subjected to tissue analysis. The morphological data showed that MSM suppressed xenograft growth. In the presence of 300 mM MSM, xenograft growth was reduced by 70% compared to the control animals (Fig. 7B). Multiple targets for MSM were analyzed *in vivo* using Western blotting and RT-PCR. We found the results from these studies to concur with the *in vitro* analysis results (Fig. 7D and E). We also performed immunohistochemical analysis of the xenograft in order to observe the expression patterns of VEGF, STAT3, IGF-1R, and STAT5b (Fig. 8A and B). These results also showed MSM can control VEGF, STAT3, IGF-1R, and STAT5b effectively.

The live cell microscopic study confirmed the ability of MSM to inhibit cell migration and *in vivo* studies with breast cancer xenograft model confirmed the ability of MSM to suppress tumor growth. Both from *in vitro* and *in vivo* analysis we confirmed that MSM had a regulatory mechanism on STAT3, STAT5b, IGF-1R, IGF-1, and VEGF. This confirmed the ability of MSM to suppress tumor initiation, growth, and metastasis. Based on all these results from our study, we strongly recommend the use of MSM as a trial drug for treating breast cancers because of its multi-targeting mechanism.

## Supporting Information

Movie S1 **Live cell video of MDA-MB 231 cell incubated in DMEM medium in the absence of MSM.**
(MP4)

Movie S2 **Live cell video of MDA-MB 231 cell incubated in DMEM medium with 200 mM MSM.**
(MP4)

Table S1 **Primers sequences used for the RT-PCR analysis.**
(DOCX)
