# Supplementary material for: Methylsulfonylmethane Suppresses Breast Cancer Growth by Down-Regulating STAT3 and STAT5b Pathways
Source: PLoS One. 2012 Apr 2;7(4):e33361. doi: 10.1371/journal.pone.0033361 (PMC3317666; doi:10.1371/journal.pone.0033361)
Supplement: Table S1 — Primers sequences used for the RT-PCR analysis. (DOCX) [file pone.0033361.s003.docx]

Table 1. Primers sequences used for the RT-PCR analysis

| Name | Sequences (5’ ->3’) | |
| --- | --- | --- |
| IGF-1R | sense | ACTATGCCGGTGTCTCTGTG |
|  | antisense | TGCAAGTTCTGGTTGTCGAG |
| VEGF | sense | AGGAGGGGAGAATCATCAGG |
|  | antisense | CAAGGCCGAGAGGGATTTTC |
| 18s | sense | CGGCTACCACATCCAAGGAA |
|  | antisense | CCGGCGTCCCTCTTAATC |
